# Incidence and predictors of brain infarction in neonatal patients on extracorporeal membrane oxygenation: an observational cohort study

**DOI:** 10.1038/s41598-022-21749-5

**Published:** 2022-10-26

**Authors:** Sarah Kopfer, Riccardo Iacobelli, Sara Wood, Caroline Lindblad, Eric Peter Thelin, Alexander Fletcher-Sandersjöö, Lars Mikael Broman

**Affiliations:** 1 ECMO Centre Karolinska, Department of Pediatric Perioperative Medicine and Intensive Care, Astrid Lindgren Children’s Hospital, Karolinska University Hospital, 171 76 Stockholm, Sweden; 2 Department of Clinical Neuroscience, Karolinska Institutet, Stockholm, Sweden; 3 Department of Neurology, Karolinska University Hospital, Stockholm, Sweden; 4 Department of Neurosurgery, Karolinska University Hospital, Stockholm, Sweden; 5 Department of Physiology and Pharmacology, Karolinska Institutet, Stockholm, Sweden

**Keywords:** Paediatric research, Cardiology, Neurology, Risk factors, Hypoxia, Neurological manifestations, Respiratory signs and symptoms, Cardiovascular diseases, Neurological disorders, Respiratory tract diseases, Infection, Inflammation

## Abstract

To determine the incidence and identify predictors of brain infarctions (BI) in neonatal patients treated with extracorporeal membrane oxygenation (ECMO). We performed a retrospective cohort study at ECMO Centre Karolinska, Stockholm, Sweden. Logistic regression models were used to identify BI predictors. Neonates (age 0–28 days) treated with veno-arterial (VA) or veno-venous (VV) ECMO between 2010 and 2018. The primary outcome was a computed tomography (CT) verified BI diagnosed during ECMO treatment. In total, 223 patients were included, 102 patients (46%) underwent at least one brain CT and 27 patients (12%) were diagnosed with a BI. BI diagnosis was associated with increased 30-day mortality (48% vs. 18%). High pre-ECMO Pediatric Index of Mortality score, sepsis as the indication for ECMO treatment, VA ECMO, conversion between ECMO modes, use of continuous renal replacement therapy, and extracranial thrombosis were identified as independent predictors of BI development. The incidence of BI in neonatal ECMO patients may be higher than previously understood. Risk factor identification may help initiate steps to lower the risk or facilitate earlier diagnosis of BI in neonates undergoing ECMO treatment.

## Introduction

Extracorporeal membrane oxygenation (ECMO) is a life-sustaining treatment for refractory severe lung and/or heart failure often used when conventional intensive care therapies fail. However, in addition to the critical condition of the patient, ECMO treatment itself is associated with significant morbidity and mortality. A common and severe complication is brain infarction (BI)^1,2^. The pathophysiology behind ECMO-related BI is likely a combination of pre-ECMO comorbidities, the associated inflammatory response and ECMO-induced hemostatic disruption^1,3–5^. The reported incidence of BI in previous studies is 4–5% and is associated with lower survival rate in neonatal cohorts^6,7^. However, these studies are registry-based, and we hypothesize that the true incidence of BI may in fact be higher, in line with our previous findings in adults treated on ECMO support^8^.

To date, outcome modelling studies on ECMO treated neonates have largely focused on risk factors associated with hemorrhagic stroke, or combined ischemic and hemorrhagic stroke^9–11^. Previous studies suggest that prematurity, pre-ECMO severity of illness, pre-ECMO cardiac arrest and use of veno-arterial (VA) ECMO may be associated with an increase of neurological adverse events in neonates during ECMO support, including BI^6,7^. Both Anton-Martin et al. and Reed et al. focused on coagulation parameters and their findings showed no differences between cases with ischemic stroke and controls^12,13^. Current data is heterogeneous and further research is needed to assess possible predictors for BI. We aimed to explore the incidence and identify predictors of CT-verified BI in neonatal patients treated with ECMO utilizing an extensive retrospective cohort.

## Methods

The study was performed in accordance with the declaration of Helsinki and approved by the Regional Ethical Review Board in Stockholm, Sweden (DNR: 2018/830-31), who waived the need for informed consent.

All neonatal patients (0–28 days of age) treated with VA or veno-venous (VV) ECMO at ECMO Centre Karolinska, Karolinska University Hospital, Stockholm, Sweden, a high-volume respiratory ECMO center treating all age groups, between January 2010 and December 2018 were eligible for inclusion.

The primary outcome was any type of BI verified by computerized tomography (CT) performed during ECMO treatment. Patients with known cerebrovascular malformations, BI, or intracranial hemorrhage (ICH) prior to ECMO initiation, as well as those treated > 12 h at another ECMO center, were excluded from the study. To reduce the influence of precipitating events, patients were also required to have been on ECMO support for at least 12 h prior to decannulation or the development of a BI, in accordance with previous studies from our center^3,8^.

Data was collected from the hospital’s digital medical charts. Possible predicting variables were recorded until discharge or CT detection of a BI. The brain CTs performed were interpreted by an experienced pediatric radiologist or neuroradiologist. Radiographic data from all CT scans performed during hospitalization and data from daily blood samples were also collected. Patients in whom no CT was performed were classified as not having developed a BI, in accordance with previous studies from our center^8^. Variables were recorded until the detection of a BI or ECMO decannulation, including coagulation variables.

Neonatal patients were cannulated via the right jugular vein (RJV) with a 13 French (Fr, outer diameter = Fr/3 mm) OriGen (OriGen Biomedical, Burladingen, Germany) dual-lumen cannula for VV support. For VA ECMO, a jugulo-carotid approach was used with an 8–12 Fr Bio-Medicus (Medtronic, Tolochenaz, Switzerland) lighthouse tip drainage cannula placed via the RJV in the mid part of the right atrium. The arterial cannula was an 8–10 Fr Bio-Medicus (Medtronic) placed into the right carotid artery. The ECMO circuit was composed of a Medos HILITE 800LT membrane lung (Medos Medizintechnik, Stolberg, Germany/Xenios AG., Heilbronn, Germany) and tubing with bonded heparin (Cortiva™, Medronic) for increased biocompatibility. Until the fall 2011 CAPS roller pump (Stöckert, Munich, Germany) was used. Since then, the Pedivas/CentriMag (Levitronix, Zurich, Switzerland) centrifugal pump console has been used.

Anticoagulation was achieved by continuous intravenous infusion of unfractionated heparin targeting an activated partial thromboplastin time (aPTT) of 1.5–2 times the upper normal limit, which was monitored at least three times daily. During treatment, a bedside ECMO specialist nurse regularly performed neurological checks, including brainstem reflexes and pupillary examinations. When a neurological event occurred (e.g., seizure, pupillary abnormalities, confusion, lowered consciousness) a brain CT scan was performed. Additional brain CT scans were also generally performed whenever the patient was referred for a thoracic or abdominal CT scan, even in the absence of neurological symptoms. In the events of a brain infarction, anticoagulation targets were not routinely altered.

Shapiro–Wilks’s test was used to test for normality of distribution. Normally distributed continuous data are presented as mean (± SD), non-parametric continuous data as median (IQR) and categorical data as numbers and fractions (%). Statistical significance level was set to p < 0.05. A univariable logistic regression model was performed with BI as the dependent variable and possible risk factors as explanatory variables. Explanatory variables that showed a trend towards significance (p < 0.1) in the univariate regression were then added to a step-down multivariable logistic regression to determine independent predictors of BI. Multiple imputation with five iterations was used to replace missing data prior to the multivariable analysis. The Markov chain Monte Carlo method was used. Missing data was present in 8 variables, accounting for a total of 2.9% of all values. Four values had > 5% missing: Pediatric Index of Mortality (PIM) score (31.4%), lactate levels (16.6%), pO_2_ (9%) and pCO_2_ (7.2% missing). In the multivariable step-down model, the least significant factor was sequentially eliminated until only significant variables remained. Analyses were conducted using SPSS (version 27.0, IBM Corp, Armonk, NY, USA) and Fig. 3 was created using ggplot2 package through the interface RStudio (RStudio, Boston, MA, USA).

### Ethics declarations

The study was performed in accordance with the declaration of Helsinki and approved by the Regional Ethical Review Board in Stockholm, Sweden (DNR: 2018/830-31), who waived the need for informed consent.

## Results

**Figure 1:**
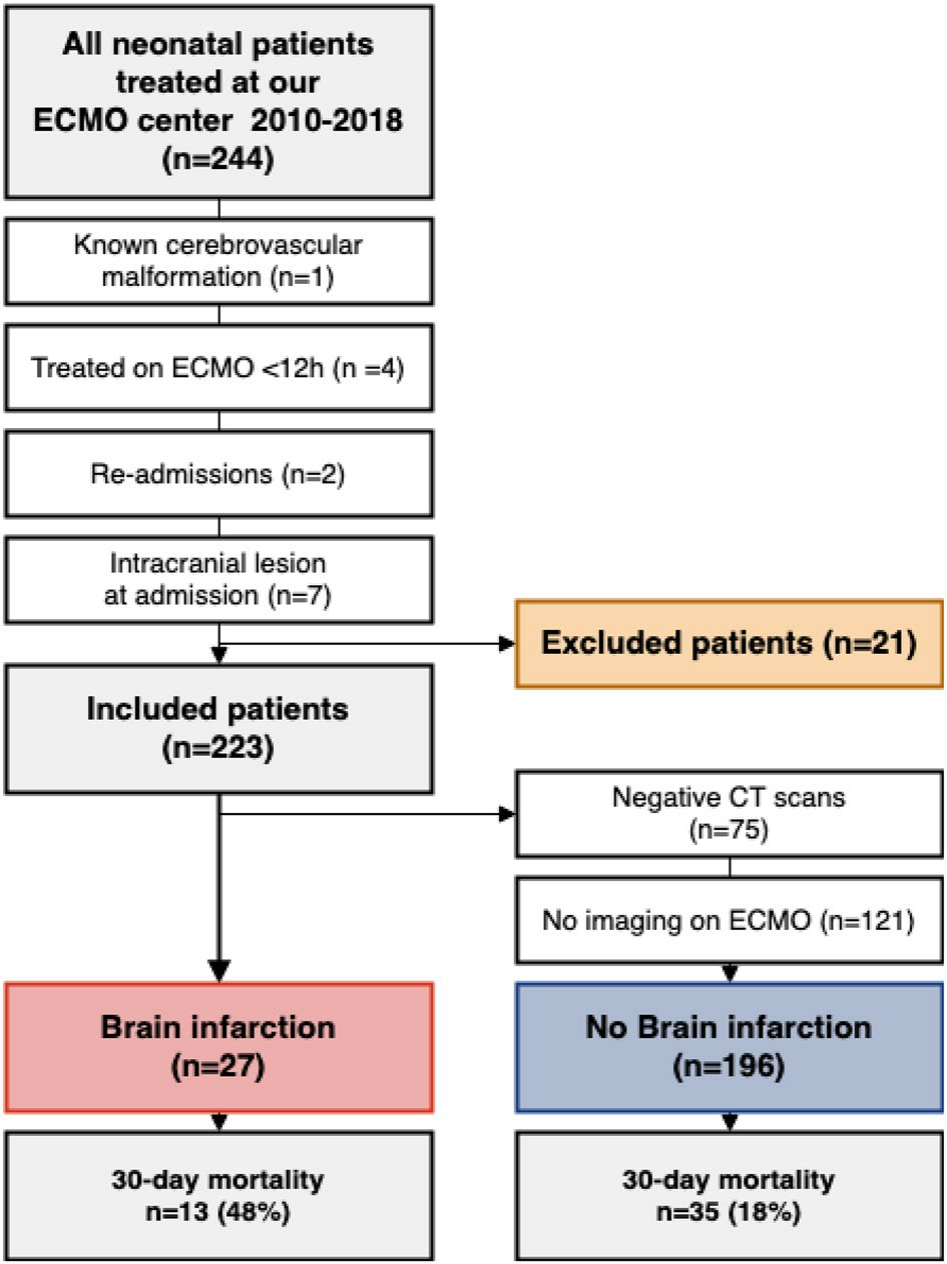
Flow-chart of the patient inclusion process and main results. *CT =* computed tomography, *ECMO* = extracorporeal membrane oxygenation, *ICH* = intracranial hemorrhage, *CNS* = central nervous system.

During the study period, 244 neonatal patients were admitted for ECMO treatment. Of these, 21 patients were excluded (Fig. 1). The most common ECMO indication was meconium aspiration syndrome (MAS) (38%), and the most common mode was VA ECMO (72%). Average age at ECMO initiation was 1 day and the average treatment length was 6 days (Supplementary Table 1).

**Figure 2:**
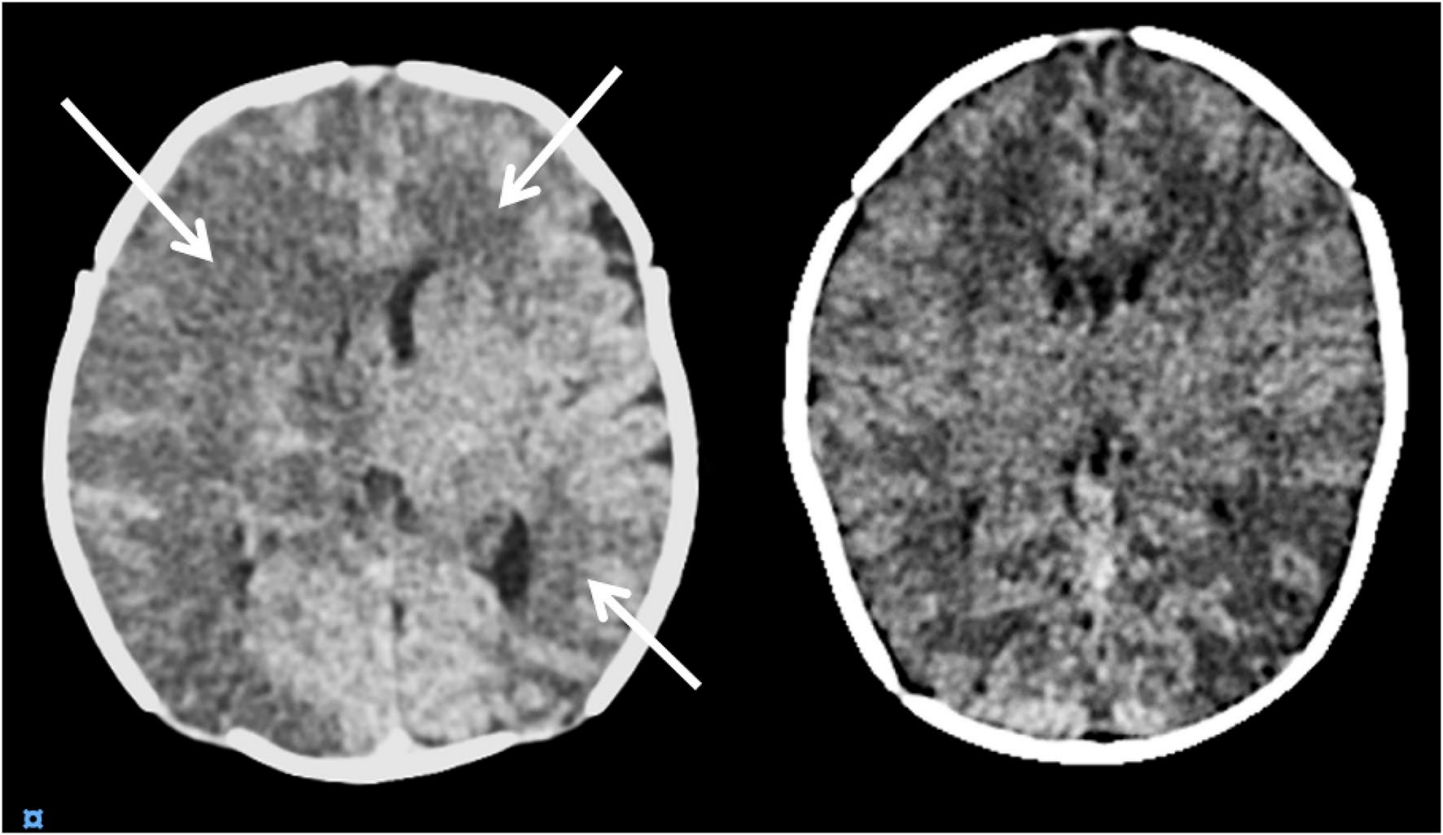
Examples of computed tomography scans of patients who developed brain infarctions during ECMO treatment. The left panel shows an almost complete right-sided thromboembolic infarction in the right middle cerebral artery territory and a smaller infarction in the left frontal and lateral occipital lobe. The right panel shows a deep brain infarction caused by hypoperfusion injury and/or possibly posterior reversible encephalopathy syndrome.

In total, 27 patients (12%) were diagnosed with a BI during ECMO treatment. Of these, 16 were believed to have been caused by thromboembolism, 10 by cerebral hypoperfusion, and 1 by a combination of both (Fig. 2). The median time from ECMO initiation to BI diagnosis was 4 days (2–9). Compared to the non-BI cohort, patients who were diagnosed with a BI had a higher 30-day (48% vs. 18%, p < 0.01) and 6-month mortality (48% vs. 20%, p < 0.01) (Table 1). Of note, 125 patients expressed no neurological symptoms and did thus not undergo CT scan during treatment and were classified as not having developed a BI. Neurological symptoms prompted a CT scan in 13 of the 27 patients with a BI, with the rest discovered by a CT performed in the absence of any neurological indication (52%).

**Table 1:** Data comparison stratified by brain infarction status.

Variable	Brain infarction (n = 27)	No brain infarction (n = 196)
Male sex	16 (60%)	110 (56%)
Gestational age (weeks)	40 (38 − 40 + 5), (2 missing, 7%)	40 (37 + 5 − 41), (5 missing, 3%)
Gestational weight (g)	3314 (± 690), (2 missing, 7%)	3446 (± 727), (5 missing, 3%)
Cardiac arrest	6 (22%)	35 (18%)
PIM (EMR%)	68 (29–87), (8 missing, 30%)	32 (19–53), (62 missing, 32%)
ABG-pH	7.14 (7.00–7.26), (2 missing, 7%)	7.20 (7.10–7.30), (15 missing, 8%)
ABG-PaCO2 (kPa)	7.5 (5.6–10.5), (2 missing, 7%)	7.7 (6.1–9.8), (14 missing, 7%)
ABG-PaO2 (kPa)	4.4 (3.2–6.5), (2 missing, 7%)	4.7 (3.4–6.5) (17 missing, 9%)
ABG-Lactate	7.8 (4.3–12.6), (7 missing, 26%)	4.1 (1.8–7.1), (30 missing, 15%)
CDH	5 (19%)	34 (17%)
ECPR	0 (0%)	3 (2%)
MAS	7 (26%)	77 (39%)
PPHN	4 (4%)	21 (11%)
Sepsis incl. septic shock	6 (22%)	17 (9%)
Other heart failure	1 (4%)	19 (10%)
Other respiratory failure	4 (15%)	25 (13%)
VA ECMO	25 (93%)	135 (69%)
Conversion	3 (11%)	5 (3%)
ECMO circuit change	10 (37%)	57 (29%)
Extracranial thrombosis	6 (22%)	11 (6%)
Cannula thrombosis	12 (44%)	123 (63%)
Extracranial bleeding	13 (48%)	47 (24%)
CRRT	25 (93%)	135 (69%)
BI detection (days)	4 (2–9)	–
Days on ECMO	7 (4–13.5)	6 (4–10)
30-Day mortality	13 (48%), (6 missing, 22%)	35 (18%), (46 missing, 23%)
6-Month mortality	13 (48%), (6 missing, 22%)	40 (20%), (46 missing, 23%)

Values are expressed as median (interquartile range), numbers (proportion) or mean (standard deviation). *ABG* = arterial blood gas, *BI* = brain infarction, *CDH* = congenital diaphragmatic hernia, *CRRT* = continuous renal replacement therapy, *ECMO =* extracorporeal membrane oxygenation, *ECPR* = extracorporeal cardiopulmonary resuscitation, *EMR%* = estimated mortality rate in percent, *MAS* = meconium aspiration syndrome, *PIM* = pediatric index of mortality, *PPHN* = persistent pulmonary hypertension in the newborn, *VA* = venoarterial.

In the univariable logistic regression predicting BI development, significant association was seen for higher pre-ECMO PIM score (p < 0.001), higher pre-ECMO arterial lactate concentration (p = 0.007), sepsis (including septic shock) as the indication for ECMO treatment (p = 0.037), VA ECMO (p = 0.021), conversion between ECMO modes (p = 0.040), continuous renal replacement therapy (CRRT) during ECMO (p = 0.021), extracranial thrombosis (p = 0.005), and extracranial bleeding (p = 0.008) (Table 2). Of these, pre-ECMO PIM score, sepsis including septic shock, VA ECMO, conversion between ECMO modes, CRRT during ECMO, and extracranial thrombosis all demonstrated independent risk association in the step-down multivariable model (Table 3).

**Table 2:** Univariable logistic regression of possible predictors for brain infarction diagnosis during ECMO treatment.

Variable	Univariable p-value
Male sex	0.758
Gestational age (days)	0.966
Gestational weight (g)	0.388
Cardiac arrest	0.583
Pre-ECMO PIM (EMR%)	**< 0.001**
Pre-ECMO ABG-pH	0.148
Pre-ECMO ABG- PaCO2 (kPa)	0.661
Pre-ECMO ABG- PaO2 (kPa)	0.132
Pre-ECMO ABG-lactate	**0.007**
Sepsis incl septic shock	**0.037**
VA ECMO	**0.021**
Conversion between ECMO modes	**0.040**
ECMO circuit change	0.398
Cannula thrombosis	0.072
Extracranial thrombosis	**0.005**
Extracranial bleeding	**0.008**
Days on ECMO	0.204
CRRT	**0.021**

Bold text highlights significant statistically significant parameters (p-value < 0.05).

*ABG* = arterial blood gas, *CI* = confidence interval, *CDH* = congenital diaphragmatic hernia, *CRRT* = continuous renal replacement therapy, *ECMO* = extracorporeal membrane oxygenation, *ECPR* = extracorporeal cardiopulmonary resuscitation, *EMR%* = estimated mortality rate in percent, *MAS* = meconium aspiration syndrome, *PIM* = pediatric index of mortality, *PPHN* = persistent pulmonary hypertension in the newborn, *VA* = venoarterial.

**Table 3:** Multivariable regression of possible predictors for brain infarction diagnosis during ECMO treatment.

Variable	Odds ratio (95% CI)	Multivariable p-value	Nagelkerke’s pseudo R^2^
Pre-ECMO PIM (EMR %)	**1.04 (1.02–1.07)**	**< 0.001**	**0.181**
Pre-ECMO ABG-lactate	1.04 (0.94–1.16)	0.468	–
Sepsis incl septic shock	**3.78 (1.13–12.62)**	**0.031**	**0.033**
VA ECMO	**6.43 (1.05–39.39)**	**0.044**	**0.069**
Conversion	**8.06 (1.12–57.86)**	**0.038**	**0.030**
Cannula thrombosis	0.49 (0.18–1.36)	0.169	–
Extracranial thrombosis	**3.94 (1.03–15.10)**	**0.045**	**0.058**
Extracranial bleeding	1.80 (0.64–5.01)	0.263	–
CRRT	**5.61 (1.14–27.69)**	**0.034**	**0.069**

Bold text highlights significant statistically significant parameters in the last step of the multivariable analysis (p-value < 0.05). All these variables were initially included in the multivariable analysis and excluded in a step-down regression model, were variables that did not meet the criteria for further inclusion, i.e., a p-value > 0.05, were excluded. Nagelkerke’s pseudo R^2^ for each of the significant variables is reported. Total pseudo R^2^ for the final multivariable model was 0.34. *ABG* = arterial blood gas, *CI* = confidence interval, *CDH* = congenital diaphragmatic hernia, *CRRT* = continuous renal replacement therapy, *ECMO* = extracorporeal membrane oxygenation, *ECPR* = extracorporeal cardiopulmonary resuscitation, *EMR%* = estimated mortality rate in percent, *MAS* = meconium aspiration syndrome, *PIM* = pediatric index of mortality, *PPHN* = persistent pulmonary hypertension in the newborn, *VA* = veno-arterial.

**Figure 3:**
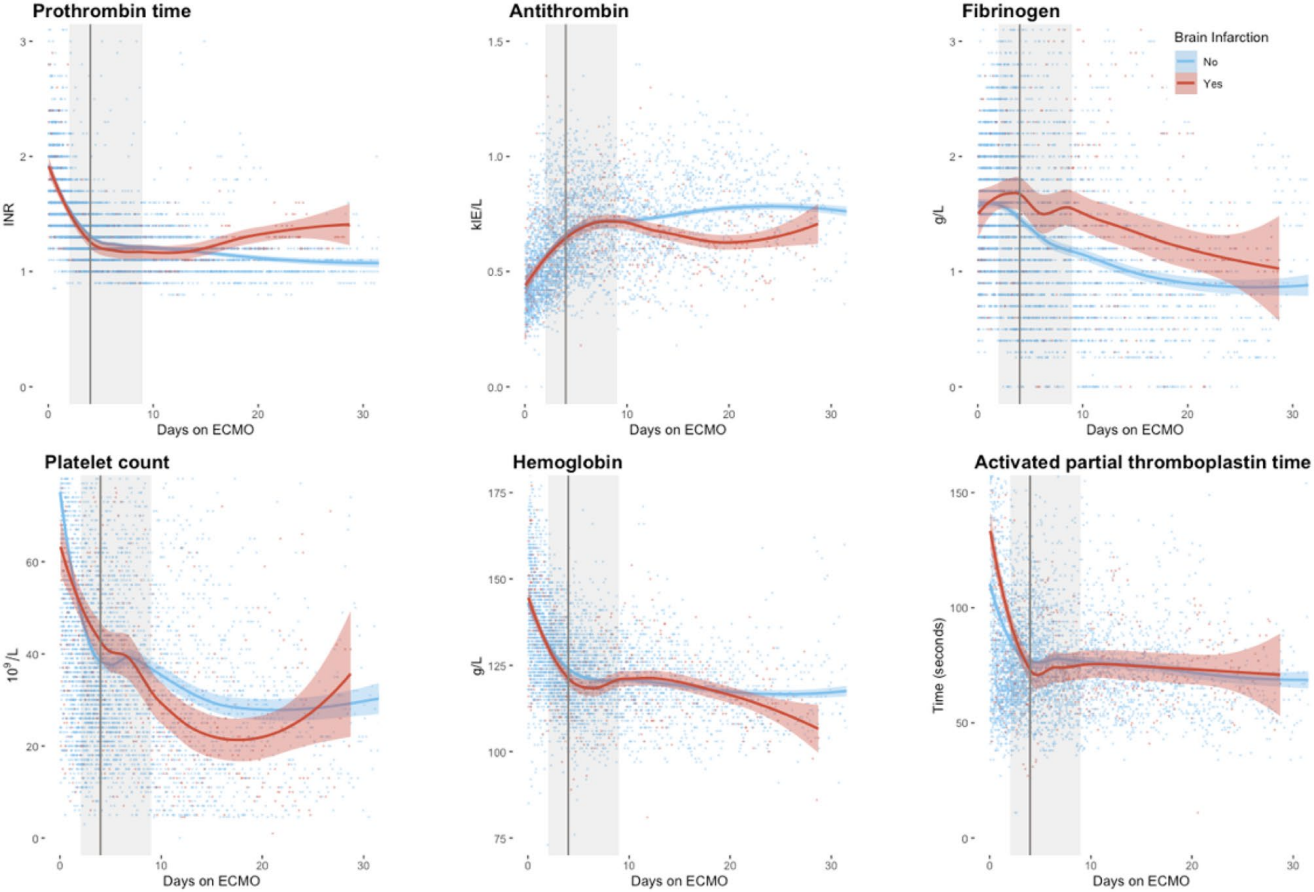
Longitudinal laboratory data. Coagulation assessment during ECMO support stratified by the presence or absence of a brain infarction. Locally weighted scatterplot smoothing curves are displayed, and the shaded area depicts their 95% confidence intervals. The vertical line represents median time to brain infarction, the shaded area represents the interquartile range.

Temporal changes in international normalized ratio (INR), antithrombin, fibrinogen, platelet count, hemoglobin and aPTT, following ECMO initiation and stratified by BI status, are presented in Fig. 3. Examined graphically, there was no obvious difference in laboratory data on ECMO initiation and throughout median or IQR timing for BI development. Patients with BI displayed a trend towards lower antithrombin, higher fibrinogen and lower platelet count towards the end of treatment.

## Discussion

In this observational cohort study, we explored possible predictors of BI in neonatal patients treated with ECMO. To the best of our knowledge, this is the largest retrospective single-center study to investigate this. Out of 223 patients, 27 (12%) developed a BI. High pre-ECMO PIM score, sepsis, VA ECMO, conversion between ECMO modes, CRRT, and extracranial thrombosis were all identified as independent predictors of BI. BI patients showed higher 30-day and 6-month mortality compared to non-BI patients.

We report a higher incidence of BI than previously reported in neonatal ECMO (4–5%)^6,7^. This may be explained by several factors. We included all BI with no distinction based on size or type, which may partly explain the discrepancy compared to a large registry study^14^. Moreover, our center has a generous radiographic policy. Head CT scans are typically performed concomitantly with extracranial CT scans, even without neurological symptoms. This is in line with previous studies from our center highlighted by the fact that 52% of the BIs in this cohort were diagnosed from a CT scan performed in the absence of symptoms^3,8^. It is plausible that some patients with known BI may have been admitted with undetected perinatal BIs, making the true incidence possibly higher as subclinical BI may go undetected during ECMO treatment^15^. On the other hand, we strive to keep patients on ECMO awake whenever possible, which may reduce the risk of patients suffering undetected clinically relevant BI, even without a CT scan.

The PIM score is a risk stratification system used to predict mortality in pediatric ICU-patients. It comprises parameters related to ICU admission and patient characteristics^16^. Previous studies of pediatric populations identified arterial pH < 7.2, need for cardiopulmonary resuscitation, and pre-ECMO metabolic acidosis as predictors of neurological complications, all of which are considered in the PIM score^7,17^. While we cannot state which aspects of the PIM score that drove the increased risk of BI in our patients, our study shows that it may predict BI development in neonatal ECMO.

Previous studies of neonates identified an association between neurological complications during ECMO and low birth weight, prematurity, low gestational age, pre-ECMO cardiac arrest, and VA ECMO^7,18^. We were only able to reproduce the significance of VA ECMO. Wien et al. performed a MRI study on neonates after ECMO support and found that patients treated with VA had a higher rate of BI compared to VV (49% vs. 29%)^19^. One possible cause behind this could be the loss of pulsatile flow, with subsequent negative effects on endothelial reactivity and cerebral autoregulation seen in VA ECMO patients^20^.

Conversion between ECMO modes was independently associated with BI development. This is in accordance with two recent studies that found conversion from VV to VA ECMO to be associated with increased complications and/or mortality in neonates and children^21,22^. We did not assess what led to conversion in this study, but a previous study from our center on adults showed that the most common reason for conversion was right ventricular failure, probably due to increased pulmonary vascular resistance^23^. One explanation for the association with BI may be the prolonged risk of exposure to brain hypoxia or thromboembolic events during the conversion. However, if the conversion was performed due to the worsened status of the patient, the BI might be explained by disease severity rather than the conversion itself. Of note, changes of circuit components during support did not influence primary outcome in the univariable analysis (Table 2). It should also be noted that the number of conversions in this study was low, 3 patients (11%) in the BI-group and 5 (3%) in the non-BI group. Thus, drawing any major conclusions concerning ECMO conversion based on our findings should be made with caution.

Among the studied ECMO indications, only sepsis proved to be an independent predictor of BI development, which is novel. However, sepsis is a known risk factor for BI development in neonatal non-ECMO populations^24^, presumably because it can lead to hemostatic disturbance, endothelial injury, and release of inflammatory mediators^15^. This may be further exacerbated by the inflammatory response caused by the exposure of blood to artificial surfaces per se in the ECMO circuit^20^. The graphical depiction of hemostatic analyses over time showed lower antithrombin, higher fibrinogen, and lower platelet count more prevalent in patients who developed a BI. Antithrombin deficiency has been linked to excessive blood clotting in non-ECMO populations^25^. Similarly, elevated levels of fibrinogen has been associated with thrombosis although causality could not be determined^26,27^. Fibrinogen is an acute phase protein and may merely reflect the association between sepsis and BI seen in our study. The latter could also explain the relationship between BI and low platelet as well as antithrombin levels seen in sepsis^28,29^. Of note, both hypoxic and thromboembolic strokes comprise BI in our study. As such, their pathophysiology differs significantly, and biochemistry may have different implication in ECMO patients’ BI development.

Requirement of CRRT during ECMO treatment was also an independent predictor of BI. Acute kidney failure was the main indication for CRRT in our study population. This finding is in line with results presented by Cengiz et al., who found that ECMO-treated pediatric patients with acute kidney injury and CRRT requirement had an increased risk for neurologic complications^17^. The association between CRRT and BI may also be explained by the increased risk of hemolysis associated with CRRT during ECMO, in which plasma free hemoglobin eliminate nitric oxide in the microcirculation leading to increased inflammation, coagulation activation, and vasoconstriction^30–32^. ECMO per se may also cause hemolysis, and moderate to severe hemolysis is known to increase mortality risks in ECMO patients about five-fold^33^.

Lastly, extracranial thrombosis demonstrated independent risk association with BI development. This predictor has not been previously studied in the neonatal population. Extracranial thrombosis may be a finding suggestive of hemostatic disruption within the ECMO circuit, the anticoagulation regime, or the severity of the indication for ECMO. Thus, it might be viewed as a surrogate marker for thrombotic tendencies. Furthermore, cannula thrombosis was not included in extracranial thrombosis. It is plausible that emboli classified as extracranial thrombosis may stem from the cannula itself. This may lead to some overlapping between the variables that further makes drawing any conclusion on these findings difficult. Of note, central venous line thrombosis was clinically observed during the study period and today we do not routinely use these catheters in neonates during ECMO.

Unfortunately, we found that longitudinal observation of coagulation parameters (Fig. 3) was very difficult to draw any conclusion from. Mostly, the confidence interval of the different parameters overlaps, until end of treatment. It is important to note that this separation occurs after the median and 75% IQR time of BI, which informs that the graph is based on fewer observations. Therefore, we refrained from making any conclusion regarding the displayed graphs.

The major limitation of this work relates to screening and diagnostics of BI. Brain Magnetic resonance imaging (MRI) is more sensitive and more specific than CT scanning for brain infarction^34^. However, it is often impossible to perform during ECMO, since the metal wire reinforced cannulas used are not MRI compatible. MRI is usually performed later in the healthcare chain. Since we are a dedicated ECMO ICU our patients are observed for about 24 h after ECMO decannulation and then transferred to another PICU or referring hospital. There the patient will be treated according to their standards and included in their follow-up programs accordingly. As it is not standard clinical practice at our center, MRI data was therefore unavailable to us. Routine CT scans were not performed on admission, indicating that some patients may have developed subclinical BI before ECMO treatment initiation. In addition, patients who did not undergo CT scans were categorized as not having developed a BI, which may have led to underestimation of BI incidence in our cohort. However, this is consistent with previous studies and likely only affected subclinical BI^35–37^. We performed a sub-group analysis where we excluded patients where no CT scan was performed (Supplementary Table 2, Supplementary Table 3). In this sub-group analysis, PIM score (p = 0.014), sepsis (p = 0.045) and pre-ECMO lactate levels (p = 0.004) were still found to be statistically significant. However, we believe that this does not accurately reflect the clinical setting and the typical ECMO population. In these patients, there was at least one indication for CT—the patient was not doing well. The result of the sub-group analysis likely reports a falsely high incidence of BI, 26.5%. On the other hand, we believe that the main result of our study may underestimate the true incidence of BI in neonatal ECMO patients may be even higher. Perhaps the true incidence lies somewhere between the 12–26.5% reported in our analysis and sub-group analysis. We included data from both VA and VV ECMO, as we believe this more accurately depicts the clinical setting of dedicated ECMO centers and is especially important when conversions between modalities are considered. PIM score data was missing for patients admitted before 2013. However, the proportion of missing data for BI and non-BI groups was largely the same, and we deemed it therefore suitable to replace the missing values using multiple imputation. Moreover, we included all neonates regardless of gestational age and used gestational age as a continuous variable, rather than separating pre- and post-term neonates concerning risk factor for neurologic injury, as there may be inherent differences in complications. Some patients treated at our center are registered and reside outside Sweden. We were unable to access data regarding BI for patients transported back for continued ECMO support. Likewise, 23% (n = 52) were lost to follow up due to transfer to other regions or countries after decannulation from ECMO. The proportions were similar in the BI and non-BI groups (22 vs. 23%).

We also performed multiple testing which introduces a risk for type I errors. We chose not to adjust for multiple comparisons as this study was only hypothesis-generating. Thus, all predictors identified need to be externally validated before definitive clinical conclusions can be drawn. In addition, most of these predictors are non-modifiable during ongoing ECMO. Nevertheless, awareness of risk factors increases the chance for recognition of subject at risk for individualized management of anticoagulation and neuromonitoring. This may limit BI frequency. Means for neuromonitoring may include lower thresholds for CT-scanning, intensified assessment of biochemistry (e.g. coagulations and biomarkers of brain injury), continuous electroencephalography, cerebral near infrared spectroscopy, ultrasound of the optic nerve sheath, and transcranial Doppler^38–40^.

## Conclusions

Brain infarction in neonatal ECMO may be more common than previously reported and is associated with increased mortality. Pre-ECMO PIM score, sepsis as ECMO indication, VA ECMO, conversion between ECMO modes, CRRT, and extracranial thrombosis were independent predictors of BI development during ECMO treatment. Prospective studies are warranted to confirm these results.

## Supplementary Information

Supplementary Table 1.

Supplementary Table 2.

Supplementary Table 3.

Supplementary Table 4.

## Data Availability

The datasets generated for quality assurance from patient charts during and/or analyzed during the current study are not made public to ensure professional secrecy but are available from the corresponding author on reasonable request.

## Abbreviations

ABG: Arterial blood gas
ACT: Active clotting time
aPTT: Activated partial thromboplastin time
BI: Brain infarction
CDH: Congenital diaphragmatic hernia,
CI: Confidence interval,
CNS: Central nervous system
CRRT: Continuous renal replacement therapy
CT: Computed tomography (CT)
ECMO: Extracorporeal membrane oxygenation
ECPR: Extracorporeal cardiopulmonary resuscitation,
EMR%: Estimated mortality rate in percent,
ICH: Intracranial hemorrhage
INR: International normalized ratio
MAS: Meconium aspiration syndrome
MVs: Missing values
PIM: Pediatric Index of Mortality
PPHN: Persistent pulmonary hypertension in the newborn,
VA: Veno-arterial.
VV: Veno-venous
