# Supplementary material for: Incidence and predictors of brain infarction in neonatal patients on extracorporeal membrane oxygenation: an observational cohort study
Source: Sci Rep. 2022 Oct 26;12:17932. doi: 10.1038/s41598-022-21749-5 (PMC9605965; doi:10.1038/s41598-022-21749-5)
Supplement: Supplementary file 1 — Supplementary Table 1. [file 41598_2022_21749_MOESM1_ESM.docx]

## Supplementary table 1: Additional data comparison stratified by brain infarction status

| **Variable** | **Brain infarction (n=27)** | **No brain infarction (n=196)** |
| --- | --- | --- |
| GA <37 weeks | 5 (19%) | 37 (19%) |
| GA Week 37-38 | 3 (11%) | 26 (13%) |
| GA Week 39-40 | 11 (41%) | 73 (37%) |
| GA Week 41 | 5 (19%) | 38 (19%) |
| GA >42 weeks | 1 (4%) | 17 (9%) |
| Admission age (days) | 1 (1 – 3) | 1 (1-3) |
| Body weight at admission (g) | 3500 (2900– 3800) | 3500 (3000 – 4000) |

Values are expressed as median (interquartile range) or numbers (proportion). Chi-square test was performed to detect difference between gestational age stratified in age groups, no difference was found (p 0.93). Abbreviations: GA = Gestational Age.
