# Supplementary material for: Incidence and predictors of brain infarction in neonatal patients on extracorporeal membrane oxygenation: an observational cohort study
Source: Sci Rep. 2022 Oct 26;12:17932. doi: 10.1038/s41598-022-21749-5 (PMC9605965; doi:10.1038/s41598-022-21749-5)
Supplement: Supplementary file 2 — Supplementary Table 2. [file 41598_2022_21749_MOESM2_ESM.docx]

## Supplementary table 2: Data comparison stratified by brain infarction status

| **Variable** | **Brain infarction (n=27)** | **No brain infarction (n=70)** |
| --- | --- | --- |
| Male sex | 16 (60%) | 36 (51%) |
| Gestational age (weeks + days) | 40 (38 – 40+5), (2 missing, 7%) | 39+1 (36 – 40), (2 missing, 3%) |
| Gestational weight (g) | 3314 (± 690), (2 missing, 7%) | 3219 (± 633), (1 missing, 1%) |
| Cardiac arrest | 6 (22%) | 14 (18%) |
| PIM score | 68 (29 – 87), (8 missing, 30%) | 38 (23 – 62), (30 missing, 43%) |
| ABG pH | 7.14 (7.00 – 7.26), (2 missing, 7%) | 7.23 (7.14 – 7.32), (8 missing, 11%) |
| ABG PaCO2 (kPa) | 7.5 (5.6 – 10.5), (2 missing, 7%) | 7.7 (6.1 – 9.8), (8 missing, 11%) |
| ABG PaO2 (kPa) | 4.4 (3.2 – 6.5), (2 missing, 7%) | 4.7 (3.4 – 6.5) (8 missing, 11%) |
| ABG lactate | 7.8 (4.3 – 12.6), (7 missing, 26%) | 4.1 (1.8 – 7.1), (13 missing, 19%) |
| CDH | 5 (19%) | 21 (30%) |
| ECPR | 0 (0%) | 3 (4%) |
| MAS | 7 (26%) | 15 (21%) |
| PPHN | 4 (4%) | 10 (14%) |
| Sepsis incl. septic shock | 6 (22%) | 5 (7%) |
| Other heart failure | 1 (4%) | 7 (10%) |
| Other respiratory failure | 4 (15%) | 9 (13%) |
| VA ECMO | 25 (93%) | 58 (83%) |
| Conversion | 3 (11%) | 4 (6%) |
| ECMO circuit change | 10 (37%) | 26 (37%) |
| Extracranial thrombosis | 6 (22%) | 7 (10%) |
| Cannula thrombosis | 12 (44%) | 40 (57%) |
| Extracranial bleeding | 13 (48%) | 26 (37%) |
| CRRT | 25 (93%) | 58 (83%) |
| BI detection (days) | 4 (2 – 9) | - |
| Days on ECMO | 7 (4 - 13.5) | 8 (5 – 17) |
| 30-day mortality | 16 (59%), (6 missing, 22%) | 19 (27%), (15 missing, 21%) |
| 6-month mortality | 16 (59%), (6 missing, 22%) | 21 (30%), (15 missing, 21%) |

#### Values are expressed as median (interquartile range), numbers (proportion) or mean (standard deviation). Abbreviations: ABG = arterial blood gas, BI = brain infarction, CDH = congenital diaphragmatic hernia, CRRT = continuous renal replacement therapy, ECMO = extracorporeal membrane oxygenation, ECPR = extracorporeal cardiopulmonary resuscitation, EMR% = estimated mortality rate in percent, MAS = meconium aspiration syndrome, PIM = pediatric index of mortality, PPHN = persistent pulmonary hypertension in the newborn, VA = venoarterial.
