# Supplementary material for: Incidence and predictors of brain infarction in neonatal patients on extracorporeal membrane oxygenation: an observational cohort study
Source: Sci Rep. 2022 Oct 26;12:17932. doi: 10.1038/s41598-022-21749-5 (PMC9605965; doi:10.1038/s41598-022-21749-5)
Supplement: Supplementary file 3 — Supplementary Table 3. [file 41598_2022_21749_MOESM3_ESM.docx]

## Supplementary table 3: Sub-group analysis including only neonatal patients in which a brain CT was performed. Univariable logistic regression.

| **Variable** | **Univariable p-value** |
| --- | --- |
| Male sex | 0.489 |
| Gestational age (days) | 0.139 |
| Gestational weight (g) | 0.528 |
| Cardiac arrest | 0.809 |
| **Pre-ECMO PIM (EMR%)** | **0.014** |
| Pre-ECMO ABG pH | 0.094 |
| Pre-ECMO ABG PaCO2 (kPa) | 0.578 |
| Pre-ECMO ABG PaO2 (kPa) | 0.573 |
| **Pre-ECMO ABG lactate** | **0.004** |
| ECMO indication: CDH | 0.257 |
| ECMO indication: ECPR | 0.999 |
| ECMO indication: MAS | 0.636 |
| ECMO indication: PPHN | 0.682 |
| ECMO indication: Other heart failure | 0.332 |
| ECMO indication: Other respiratory failure | 0.800 |
| **Sepsis incl septic shock** | **0.045** |
| VA ECMO | 0.465 |
| Conversion between ECMO modes | 0.366 |
| ECMO circuit change | 0.992 |
| Cannula thrombosis | 0.122 |
| Extracranial thrombosis | 0.122 |
| Extracranial bleeding | 0.323 |
| Days on ECMO | 0.366 |
| CRRT | 0.235 |

#### Bold text highlights significant statistically significant parameters (p-value <0.05). Abbreviations: ABG = arterial blood gas, CI = confidence interval, CDH = congenital diaphragmatic hernia, CRRT = continuous renal replacement therapy, CT = computed tomography, ECMO = extracorporeal membrane oxygenation, ECPR = extracorporeal cardiopulmonary resuscitation, EMR% = estimated mortality rate in percent, MAS = meconium aspiration syndrome, PIM = pediatric index of mortality, PPHN = persistent pulmonary hypertension in the newborn, VA = venoarterial.
