# Supplementary material for: Incidence and predictors of brain infarction in neonatal patients on extracorporeal membrane oxygenation: an observational cohort study
Source: Sci Rep. 2022 Oct 26;12:17932. doi: 10.1038/s41598-022-21749-5 (PMC9605965; doi:10.1038/s41598-022-21749-5)
Supplement: Supplementary file 4 — Supplementary Table 4. [file 41598_2022_21749_MOESM4_ESM.docx]

## Supplementary table 4: Data comparison stratified by neurological symptoms as CT indication

| **Variable** | **Neurological symptoms* (n=36)** | **No neurological symptom* (n=187)** |
| --- | --- | --- |
| Male sex | 16 (60%) | 102 (55%) |
| Gestational age (weeks) | 40 (37+2 - 40+6) | 40 (37+6 - 41) |
| Gestational weight (g) | 3431 (± 723) | 3455 (± 731) |
| Cardiac arrest | 6 (22%) | 33 (18%) |
| PIM (EMR%) | 50 (19 - 70) | 32 (20 - 54) |
| ABG-pH | 7.20 (7.02 - 7.32) | 7.20 (7.09 - 7.30) |
| ABG-PaCO2 (kPa) | 7.71 (6.1 - 10.5) | 7.69 (6.0 - 9.8) |
| ABG-PaO2 (kPa) | 4.4 (3.2 - 6.5) | 4.7 (3.4 - 6.5) |
| ABG-Lactate | 5.2 (2.15 – 10.0) | 4.1 (1.8 - 7.4) |
| CDH | 9 (25%) | 30 (16%) |
| ECPR | 1 (3%) | 2 (1%) |
| MAS | 7 (19%) | 74 (40%) |
| PPHN | 3 (8%) | 22 (12%) |
| Sepsis incl. septic shock | 5 (14%) | 18 (10%) |
| Other cardiac failure | 3 (8%) | 17 (9%) |
| Other respiratory failure | 5 (14%) | 24 (13%) |
| VA ECMO | 31 (86%) | 129 (69%) |
| Conversion of ECMO mode | 3 (8%) | 5 (3%) |
| ECMO circuit change | 14 (39%) | 53 (28%) |
| Extracranial thrombosis | 5 (14%) | 12 (6%) |
| Cannula thrombosis | 20 (56%) | 115 (61%) |
| Extracranial bleeding | 19 (53%) | 41 (22%) |
| CRRT | 32 (89%) | 128 (68%) |
| Brain infarction | 16 (44%) | 11 (6%) |

Values are expressed as median (interquartile range), numbers (proportion) or mean (standard deviation). Abbreviations: ABG = arterial blood gas, BI = brain infarction, CDH = congenital diaphragmatic hernia, CRRT = continuous renal replacement therapy, ECMO = extracorporeal membrane oxygenation, ECPR = extracorporeal cardiopulmonary resuscitation, EMR% = estimated mortality rate in percent, MAS = meconium aspiration syndrome, PIM = pediatric index of mortality, PPHN = persistent pulmonary hypertension in the newborn, VA = venoarterial

*Neurological symptoms as described in this table referrers to symptoms that lead to a CT scan: symptom that occurred after an incidental CT finding are not included.
